# Isolation and Characterization of *Bacillus velezensis* Strain P2-1 for Biocontrol of Apple Postharvest Decay Caused by *Botryosphaeria dothidea*

**DOI:** 10.3389/fmicb.2021.808938

**Published:** 2022-01-04

**Authors:** Hongbo Yuan, Bingke Shi, Li Wang, Tianxiang Huang, Zengqiang Zhou, Hui Hou, Hongtao Tu

**Affiliations:** ^1^Zhengzhou Fruit Research Institute, Chinese Academy of Agricultural Sciences, Zhengzhou, China; ^2^Key Lab of Horticultural Plant Biology, Ministry of Education, and College of Plant Science and Technology, Huazhong Agricultural University, Wuhan, China

**Keywords:** apple ring rot, *Botryosphaeria dothidea*, *Bacillus velezensis*, biological control, postharvest quality

## Abstract

*Botryosphaeria dothidea* causes apple ring rot, which is among the most prevalent postharvest diseases of apples and causes significant economic loss during storage. In this study, we investigated the biocontrol activity and possible mechanism of *Bacillus velezensis* strain P2-1 isolated from apple branches against *B. dothidea* in postharvest apple fruit. The results showed strain P2-1, one of the 80 different endophytic bacterial strains from apple branches, exhibited strong inhibitory effects against *B. dothidea* growth and resulted in hyphal deformity. *B. velezensis* P2-1 treatment significantly reduced the ring rot caused by *B. dothidea*. Additionally, the supernatant of strain P2-1 exhibited antifungal activity against *B*. *dothidea*. Re-isolation assay indicated the capability of strain P2-1 to colonize and survive in apple fruit. PCR and qRT-PCR assays revealed that strain P2-1 harbored the gene clusters required for biosynthesis of antifungal lipopeptides and polyketides. Strain P2-1 treatment significantly enhanced the expression levels of pathogenesis-related genes (*MdPR1* and *MdPR5*) but did not significantly affect apple fruit qualities (measured in fruit firmness, titratable acid, ascorbic acid, and soluble sugar). Thus, our results suggest that *B. velezensis* strain P2-1 is a biocontrol agent against *B. dothidea*-induced apple postharvest decay. It acts partially by inhibiting mycelial growth of *B. dothidea*, secreting antifungal substances, and inducing apple defense responses.

## Introduction

Apple ring rot, caused by *Botryosphaeria dothidea*, is one of the most prevalent postharvest diseases affecting apples. It results in significant yield and quality losses during storage (Tang et al., 2012; Jurick et al., 2013; Zhang et al., 2016; Huang et al., 2021). Additionally, *B. dothidea* can infect apple tree branches or trunks during their growth stage, causing stem canker and tree death (Phillips et al., 2005; Xue et al., 2021). Apple ring rot caused by *B. dothidea* is difficult to control due to the latent infection characteristic (Liu et al., 2011). This disease is primarily controlled through the use of fungicides but environmental and food safety concerns severely limit their use. Furthermore, several fungicide-resistant isolates of *B. dothidea* have been frequently reported in recent years as a result of the long-term use of pesticides (An et al., 2016; dos Santos et al., 2019; Wang L. et al., 2021). Therefore, alternative and environmentally friendly methods of controlling this disease are urgently needed.

Biological control of apple ring rot disease with endophytes is an alternative method with friendly and non-toxic characteristics. A wide range of microorganisms, including *Streptomyces rochei, Meyerozyma guilliermondii*, and *Bacillus* spp., have been identified as effective in the biological control of apple ring rot disease (Chen et al., 2016; Zhang et al., 2016; Huang et al., 2021). For example, yeast *M. guilliermondii* strain Y-1 isolated from grape skins demonstrated antifungal activity against *B. dothidea* by inhibiting spore germination and mycelial growth. Furthermore, strain Y-1 was found to be capable of inducing a series of defense responses and confer host resistance (Huang et al., 2021). By stimulating a series of defense mechanisms, *S. rochei* strain A-1, isolated from healthy apple fruit can effectively inhibit postharvest decay of apples caused *by B. dothidea* (Yong et al., 2014; Zhang et al., 2016). *Bacillus* spp. is a species that has been isolated and studied extensively as a biological control agent. At present, *Bacillus* spp. including *B. subtilis* strain 9407 and *B. amyloliquefaciens* strain PG12 have been reported to possess antagonistic activity against *B. dothidea* (Chen et al., 2016; Fan et al., 2017). Several strains of *B. velezensis* have also been reported to act as biocontrol agents, preventing plant diseases and promoting plant growth. For instance, *B. velezensis* strain ZW-10 exhibited activity against the *Magnaporthe oryzae*, via secondary metabolites (Chen et al., 2020). It has been reported that *B. velezensis* strain 83 from the mango tree phyllosphere is capable of controlling anthracnose and promoting plant growth (Balderas-Ruíz et al., 2020). *B. velezensis* strain HC6 showed antimicrobial activity against the *Listeria monocytogenes*, by producing lipopeptide surfactin (Liu et al., 2020). Recent reports indicated that *B. velezensis* strains Lle-9 and D4 possessed antifungal activity against a variety of pathogenic fungi, including *B. dothidea* (Khan et al., 2020; Liu et al., 2021). However, whether *B. velezensis* is effective against *B. dothidea* in apples and the underlying mechanisms remain unknown.

In this study, 80 bacterial isolates were obtained from the branches of apple variety “Kitanosach,” which were highly resistant to *B. dothidea*. Among them, a *B. velezensis* strain P2-1 exhibited strong inhibition activity against the growth of *B. dothidea* and apple postharvest decay caused by *B. dothidea*. Furthermore, *B. velezensis* strain P2-1 was capable to suppress apple ring rot caused by a fungicide-resistant strain. Additionally, the ability of *B. velezensis* strain P2-1 to colonize apple fruit wounds was assessed and the possible biocontrol mechanism of strain P2-1 against *B. dothidea* was explored.

## Materials and Methods

### Pathogenic Fungal Isolates

The pathogens *B. dothidea*, *Valsa mali*, *V. pyri*, and *Colletotrichum gloeosporioides* strains were used in this study and stored at Zhengzhou Fruit Research Institute, Chinese Academy of Agricultural Sciences, Zhengzhou, China (Wang L. et al., 2021; Yuan et al., 2021). *B. dothidea* strain Bd220 is a carbendazim-sensitive isolate, while *B. dothidea* strain Bd7 is a carbendazim-resistant isolate bearing an E198A point mutation in the ß–tubulin gene (GAG-GCG) (Wang L. et al., 2021). The fungal strains used in this study were cultured in potato dextrose agar (PDA) (Potato extracts 200 g L^–1^, Glucose 2 g L^–1^, Agar 15 g L^–1^) and grow at 25°C.

### Isolation of Endophytic Bacteria Strains

One-year-old branches (about 1 cm in diameter) without any disease symptoms were collected from apple tree (variety named “Kitanosach”) for endophytic bacteria isolation at Zhengzhou, Henan province, China in July 2021. “Kitanosach” is a high resistant variety to *B. dothidea* (Hou et al., 2017). The endophytic bacteria were isolated according to a previous study with some modifications (Yuan et al., 2021). In brief, the bark of apple branches was peeled off and cut into small pieces (5 mm × 5 mm) and put into 75% ethyl alcohol for 1 min. The small pieces were then placed into 1% sodium hypochlorite for 5 min. After disinfection, the small pieces were washed with sterilized ddH_2_O five times and put onto sterilized filter paper to absorb water. Subsequently, the small pieces were transferred to 1 mL of ddH_2_O containing a 0.2 cm-diameter steel ball, and shaking the tube for 1 min at 4,000 rpm in a grinder (SCIENTZ-48, Ningbo city, China). Next, 100 μL of supernatant was put onto NA medium (Peptone 10 g L^–1^, Beef extract 3 g L^–1^, Sodium chloride 5 g L^–1^, Agar 15 g L^–1^). The plates were incubated at 25°C for 2–3 days, after which emerged bacteria colonies were sub-cultured onto new plates. After purification, the endophytic bacteria were used to test the antifungal activity against *B. dothidea*.

### Dual Culture Test for Screening Potential Biocontrol Bacterium

In total, 80 different endophytic bacteria strains were isolated from the branches of a healthy apple tree and were screened for antagonistic activity against *B. dothidea* strain Bd220 by dual culture test based on a pervious study with some modifications (Yuan et al., 2021). Briefly, endophyte bacteria isolates were cultivated overnight in LB broth (Peptone 10 g L^–1^, Yeast extract 5 g L^–1^, Sodium chloride 10 g L^–1^) at 200 rpm, 28°C and 3 μL of bacteria were placed at each side 2 cm from the center on PDA medium on Petri dishes. Meanwhile, a mycelial plug (5 mm in diameter) of the tested pathogenic fungi was inoculated at the center of PDA plate. Plates were incubated at 25°C for 6 days. Then, the colony diameter of the pathogenic fungal isolate was measured using vernier calipers. The negative control was just inoculated with the pathogenic fungal isolate. The test was carried out three times, and each treatment had three replicates. Inhibition = (colony diameter of control-colony diameter of treated)/(colony diameter of control) × 100%. After the preliminary screening, the antagonistic strains were further tested antifungal activity against *V. mali*, *C. gloeosporioides* and *V. pyri* strains.

An ultra-depth three-dimensional microscope (KEYENCE, Japan) was used to assess the morphological characteristics of hyphae at 2 days of dual culture. Three replicates were analyzed per treatment, with a minimum of 10 hyphae per replicate. The assay was repeated two times.

### Antagonistic Strain Identification

The endophytic bacteria strain P2-1 was identified via morphological and molecular identification. The physiological and biochemical characters of strain P2-1 were carried out according to a previous report (Holt et al., 1994). For molecular identification, total DNA of P2-1 isolate was extracted with a DNA extraction kit (Omega, Guangdong, China), and the 16S rDNA, *gyrA* and *ropB* sequences were amplified with appropriate primers in Supplementary Table 1 (Rooney et al., 2009; Fan H. et al., 2016). All PCR reactions were conducted in a reaction containing 1.5 μL of 10 × Taq buffer, 1 μL of 2.5 mM dNTPs, 1 μL of 100 mM Mg^2+^, 0.25 μL of 5 U μL^–1^ Taq DNA polymerase, 0.25 μL of each primer (10 μM), 1 μL of 10 ng μL^–1^ bacteria DNA, and ddH_2_O to a final 20 μL volume. Thermo cycler settings were: 3 min at 94°C; 30 cycles of 94°C for 30 s, 56°C for 30 s, and 72°C for 50 s; 72°C for 5 min. PCR products were sequenced by Bgi Genomics Co., Ltd., Beijing, China, and the resultant sequences were blasted against the NCBI nucleotide collection database. Highly homologous sequences were selected for multiple sequence alignment with the MEGA 7.0 software. A phylogenetic tree was constructed via a Neighbor-Joining approach, with 1,000 replicate Bootstrap analyses being used to calculate node support.

### Assessment of Supernatant of Antagonistic Strain on *Botryosphaeria dothidea* Mycelial Growth

A bacterial colony of strain P2-1 was added to the 100 mL of LB broth in a shaker at 200 rpm for 2 days at 28°C. The samples were then centrifuged for 10 min at 5,000 rpm and the obtained supernatant was passed through a filter with a 0.22 μm pore size to remove remaining bacterial cells and get the supernatant. PDA medium was mixed with different supernatant volumes to yield PDA, containing 1, 2, 5, or 10% supernatant. Each plate was inoculated with a *B. dothidea* plug (5 mm in diameter). *B. dothidea* colony diameters were measured after a 6-day inoculation period, with untreated PDA medium serving as a control. This assay was repeated three times, with three replicates each time.

### Biocontrol Activity of *Bacillus velezensis* Strain P2-1 Against *Botryosphaeria dothidea* in Apple Fruit

The activity of strain P2-1 against *B. dothidea* in apple fruit (Gala) was determined according to a previous report (Huang et al., 2021). In brief, two wounds (3 mm deep and 5 mm wide) were punctured with a sterile borer. Each wound was treated with 40 μL of strain P2-1 cell suspension (1 × 10^8^ CFU mL^–1^) or supernatant. Sterile water or fungicide carbendazim (0.8 g L^–1^) (Tianjin Hanbang Plant Protective Agent Co., Ltd., Tianjing, China) treated apples were used as negative and positive controls, respectively. Following the 24 h of incubation at 25°C with a relative humidity of 85%, the apple fruit wounds were inoculated with *B. dothidea* mycelial plugs. Disease lesion length was measured using vernier calipers at 3 and 5 days post inoculation (dpi). The assays were repeated three times, with 10 inoculation sites per experiment. Disease incidence was calculated as follows: number of disease sites/total number of inoculation sites × 100%.

### Colonization of *Bacillus velezensis* Strain P2-1 in Apple Fruit Wounds

Apple fruits (Gala) wounds were punctured with a sterile borer, according to the same procedure mentioned above, following the application of 40 μL of strain P2-1 cell suspension (1 × 10^8^ CFU mL^–1^) to each wound. The fruits were then incubated at 20°C. We collected the wounded tissue (about 0.6 g) at 0 (3 h after inoculation), 1, 2, 3, 4, 5, and 6 days post inoculation, respectively. The wounded tissue was then ground by using a grinder as the same procedure mentioned above. Serial 10-fold dilution was prepared and each dilution (0.1 mL) was put onto NA medium on Petri dishes. After 2 days of incubation at 28°C, the plates were counted for bacterial colonies. The experiment was repeated twice, with three replicates each time.

### Efficacy of *Bacillus velezensis* Strain P2-1 in Reducing Apple Fruit Natural Decay Development

Healthy apple fruits (Gala) were harvested from an orchard in Xinxiang, Henan province, China, which contained over 10-year-old trees that were severely infected with *B. dothidea*. Apple fruits were inoculated with strain P2-1, by dipping them for 2 min in the cell suspension (1 × 10^8^ CFU mL^–1^) or sterile distilled water and then dried. The treated fruits were kept in a transparent plastic box at 20°C with a relative humidity of 85%. The number of fruits with rot disease was weekly recorded. The experiment was repeated twice and each treatment consisted of three replicates, with ten fruit samples in each replicate. The percentage of disease incidence was calculated as follows: Number of rotted fruits/total number of fruits × 100%.

### Effects of *Bacillus velezensis* Strain P2-1 on Postharvest Quality Parameters of Apple

To assess the effect of strain P2-1 on the postharvest quality of apple fruits (Gala), freshly harvested fruits were treated and stored as previously described. Four parameters were determined: (i) fruit firmness, (ii) ascorbic acid content, (iii) titratable acid content, and (iv) soluble sugar content. The firmness of each apple was determined at three points along the equatorial region, using the GY-1 Texture Analyzer equipped with a 5 mm diameter flat probe (TOP, Beijing, China). Ascorbic acid was measured according to a previous study (Özden and Bayindirli, 2002). Titratable acid concentrations were determined by titration with 0.1 N NaOH to a pH of 8.1 (Wright and Kader, 1997). Soluble sugar was assessed using the fehling reagent titration method (Guo et al., 2019).

### Detection of Antibiotic Biosynthesis Genes in *Bacillus velezensis* Strain P2-1

The total genomic DNA of strain P2-1 was extracted using a DNA extraction kit (Omega, Guangdong, China). Specific genes associated with the biosynthesis of an individual antibiotic in bacteria were amplified using the primers indicated in Supplementary Table 1. The PCR products were then electrophoretically separated and sequenced. qRT-PCR was used to assess the expression levels of antibiotic biosynthesis genes in strain P2-1. The total RNA was extracted using the RNA extraction kit (Omega, Guangdong, China) from 2-day-old cultures (grown in LB broth). The Prime Script™ RT Reagent Kit (Omega, Guangdong, China) was used to synthesize first-strand cDNA from 1 μg of total RNA. qRT-PCR was performed using a Light Cycler^®^ 96 PCR Detection System (Roche, Germany) with a ChemoHS qPCR kit (Monad, Suzhou, China) following the manufacturer’s protocol. Supplementary Table 1 includes the primers for qRT-PCR. The 16S ribosomal RNA gene was used as a reference gene (Yu et al., 2015), and relative gene expression was normalized using the 2^–^^ΔΔ^ ^Ct^ method (Livak and Schmittgen, 2001). All of the experiments were carried out twice, with at least three biological replicates each time.

### Effect of *Bacillus velezensis* Strain P2-1 on Induction of Defense-Related Gene Expression

Healthy apple fruits (Gala) were evenly sprayed with strain P2-1 cell suspension (1 × 10^8^ CFU mL^–1^) for total RNA extraction. As a control, sterile distilled water treatment was used. Total RNA was extracted at 0, 24, and 48 h post inoculation (hpi), using RNA extraction kit (Omega, Guangdong, China). The transcript levels of pathogenesis-related protein (*PR*) genes *MdPR1* and *MdPR5* were measured with specific primers in Supplementary Table 1. Elongation factor 1-a (*EF1a*) was used as a reference gene. This assay was repeated two times, with three replicates each time.

## Results

### Isolation and Screening of Antagonistic Bacteria

**FIGURE 1:**
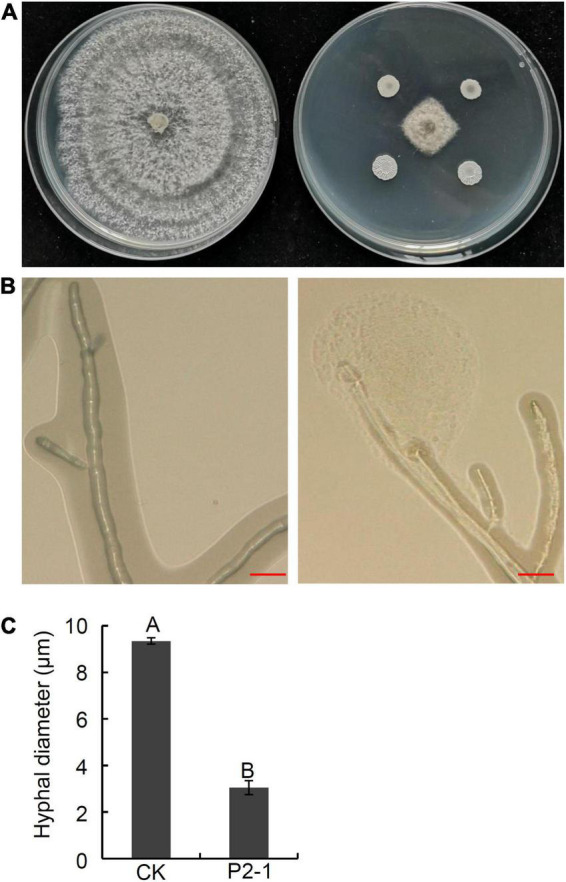
Inhibition of strain P2-1 on hyphal growth of *B. dothidea*. **(A)** Antifungal activity of strain P2-1 against *B. dothidea*. Left: *B. dothidea* CK; Right: *B. dothidea* at the present of strain P2-1. The pictures were taken at 6 days after inoculation. **(B)** Effect of strain P2-1 on hyphal morphology of *B. dothidea*. Left: *B. dothidea* CK; Right: *B. dothidea* at the present of strain P2-1. The picture was taken at 2 days after antagonism with strain P2-1 using an ultra-depth three-dimensional microscope. Bar = 20 μm. **(C)** Hyhal diameter of *B. dothidea* after antagonism with strain P2-1. Values are means ± SD from three replicates. Different letters indicate significant differences between different treatments according to Student’s *t*-test (*P* < 0.01).

In a previous work, we tested 189 apple germplasms for resistance to *B. dothidea* and discovered that two varieties, “Kitanosach” and “Qinguan,” demonstrated great resistance to ring rot disease caused by *B. dothidea* (Hou et al., 2017). In this study, endophytic bacterial strains were isolated from the bark of branches of “Kitanosach.” In total, 80 bacterial isolates were obtained and screened in the dual culture test for potential antagonistic activity against *B. dothidea*. Only three strains exhibited significant antagonistic activity against *B. dothidea*, with strain P2-1 showing enhanced inhibition of approximately 73.3% of *B. dothidea* mycelial growth (Figure 1A and Table 1). The inhibition zone of *B. dothidea* mycelial growth by strain P2-1 was of 8.6 mm. The antagonistic effect of strain P2-1 on *B. dothidea* hyphae characteristics was further assessed by microscopic observations. The results indicated that, in comparison to normal hyphae, *B. dothidea* hyphae after antagonism with the strain P2-1 exhibited abnormal stretching with deformity and a protoplast-like ball at the end of hyphae (Figure 1B). Further statistic results showed a significantly smaller average hyphal diameter of *B. dothidea* under the stress of strain P2-1 than that of control (Figure 1C).

**TABLE 1:** The inhibition of strain P2-1 against phytopathogenic fungi.

Phytopathogenic fungi	Inhibition (%)
*Botryosphaeria dothidea*	73.3 ± 2.4
*Valsa mali*	73.2 ± 1.0
*Colletotrichum gloeosporioides*	65.3 ± 1.3
*Valsa pyri*	65.2 ± 3.3

*Data represents the mean ± SD of three biological replicates.*

In addition, strain P2-1 was able to significantly inhibit the three other important fruit pathogens *V. mali*, *C. gloeosporioides*, and *V. pyri*, with respective inhibition of 73.2, 65.3, and 65.2% (Table 1). These results indicated that strain P2-1 possessed broad-spectrum antagonistic activity *in vitro*.

### Identifying the Bacterial Strain P2-1

The results of biochemical and physiological testing of strain P2-1 are summarized in Supplementary Table 2. The bacterium strain P2-1 was identified as a gram-positive strain. Positive results were obtained for VP, citrate, methyl red, V-general, nitrate reductase, starch hydrolysis, and gelatin liquefaction tests (Supplementary Table 2). Strain P2-1 could grow on media containing glucose, xylose, or mannitol as a carbon source. These features were similar to those found in *Bacillus* sp.

**FIGURE 2:**
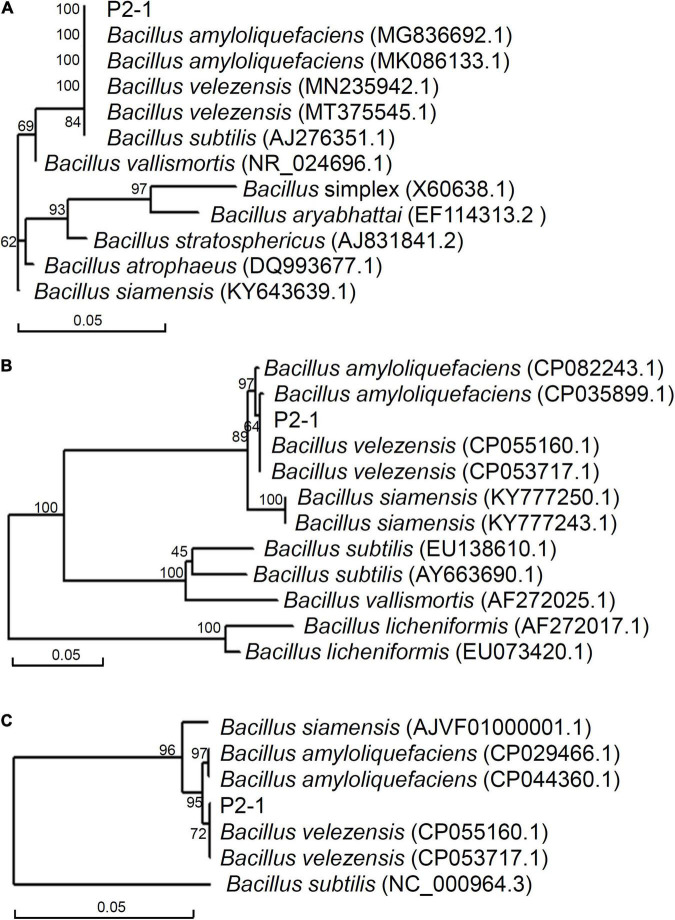
Phylogenetic analysis of strain P2-1 and its relatives based on 16 S rDNA **(A)**, *gyrA* **(B)**, and *ropB* **(C)**.

Partial 16 S rDNA sequence from strain P2-1 was amplified and used to construct a phylogenetic tree with closely related sequences. It was shown that strain P2-1 shared a branch with *B. velezensis*, *B. amyloliquefaciens*, and *B. subtilis* strains (Figure 2A). To further identify the species of strain P2-1, phylogenetic trees based on partial *gyrA* and *ropB* sequences were similarly constructed. The results indicated that strain P2-1 was most closely related to *B. velezensis* (Figures 2B,C). Therefore, strain P2-1 was identified as *B. velezensis* (16S rDNA accession number: OL314749; gyrA accession number: OL345592; ropB accession number: OL345593).

### Efficacy of *Bacillus velezensis* Strain P2-1 in Controlling Ring Rot Caused by *Botryosphaeria dothidea* in Apple

**FIGURE 3:**
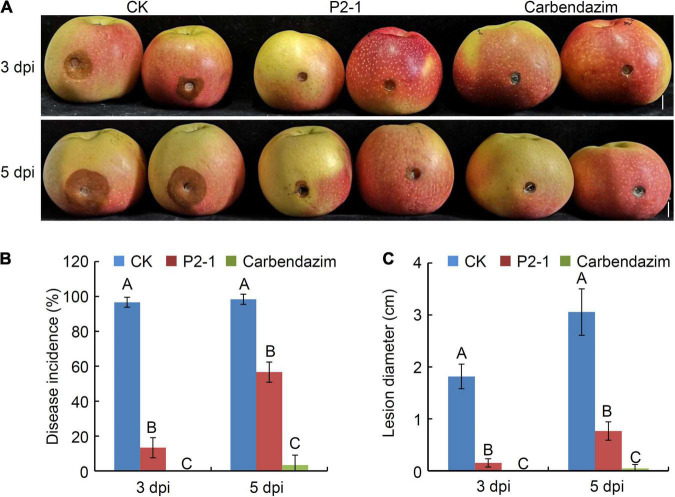
Suppression of *B. velezensis* strain P2-1 cell suspension on apple ring rot caused by *B. dothidea*. **(A)** Strain P2-1 cell suspension antagonized apple ring rot. Treatment with sterile distilled water or carbendazim was used as the negative and positive control, respectively. Bar = 1 cm. **(B)** Statistical analysis of the disease incidence. **(C)** Statistical analysis of disease lesion diameter. Each data represents the mean ± SD of three replicates. Disease lesion length and disease incidence were measured at 3 and 5 days post inoculation (dpi), respectively. Letters above the bars indicate statistical significance and different letters indicate significant different means (*p* < 0.01) based on Student’s *t*-test.

To investigate the efficacy of strain P2-1 strain in controlling the apple ring rot associated with *B*. *dothidea*, apple fruits were treated with strain P2-1 cell suspension and inoculated with *B*. *dothidea*. Three days after *B. dothidea* inoculation, the control apple fruits developed a brown lesion around the inoculation site, whereas the apple fruits treated with strain P2-1 cell suspension showed almost no disease symptom (Figure 3A). At 5 days post *B*. *dothidea* inoculation, strain P2-1 cell suspension still exhibited significant inhibition activity against disease development (Figure 3A). The disease incidence was reduced to 56.7%, compared to the control treated fruit (which was equivalent to 98.3%) (Figure 3B). As a positive control, apple fruits treated with carbendazim remained disease-free (Figure 3A). Additionally, when apple fruits were treated with strain P2-1 cell suspension, the average lesion diameter was significantly reduced when compared to the control (Figure 3C). Thus, these findings indicated that a suspension of P2-1 cells was capable of suppressing the incidence and severity of apple ring rot caused by *B. dothidea*.

### *Bacillus velezensis* Strain P2-1 Supernatant Mediated Suppression of *Botryosphaeria dothidea*

**FIGURE 4:**
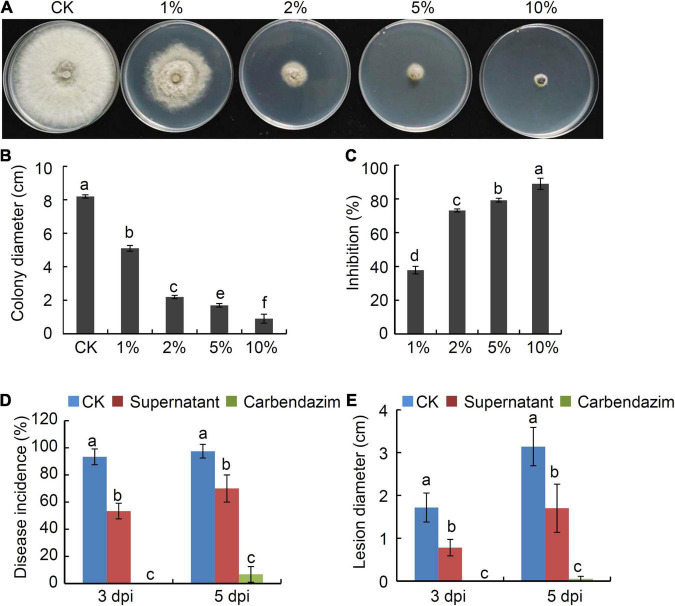
Suppression of *B. velezensis* strain P2-1 supernatant on *B*. *dothidea*. **(A)** Inhibition of strain P2-1 supernatant on mycelial growth of *B*. *dothidea*. **(B)** Statistical analysis of colony diameter. **(C)** Inhibition rate of strain P2-1 supernatant on mycelial growth *B*. *dothidea.* **(D)** Effect of strain P2-1 supernatant on apple ring rot disease incidence. **(E)** Effect of strain P2-1 supernatant on apple ring rot disease lesion diameter. Each data represents the mean ± SD of three replicates. Letters above the bars indicate statistical significance and different letters indicate significant different means (*p* < 0.05) based on Student’s *t*-test.

To determine whether strain P2-1 supernatant also possessed antagonistic activity against *B. dothidea*, we collected supernatant of strain P2-1 and added it into PDA medium to yield PDA containing 1, 2, 5, or 10% of strain P2-1 supernatant. The dual culture assay revealed that strain P2-1 supernatant suppressed *B. dothidea* mycelial growth significantly (Figures 4A,B). The inhibition of *B*. *dothidea* by 1, 2, 5 and 10% of strain P2-1 supernatant was 37.8, 73.2, 79.3, and 89.0%, respectively (Figure 4C).

Additionally, the efficiency of strain P2-1 supernatant was studied in preventing ring rot triggered by *B. dothidea* in apple fruit. The results showed that strain P2-1 supernatant significantly reduced *B. dothidea*-caused apple ring rot disease. When compared to the control, strain P2-1 supernatant dramatically reduced disease incidence and average lesion diameter (Figures 4D,E). These findings suggested that the strain P2-1 supernatant has antifungal efficacy toward *B. dothidea*.

### *Bacillus velezensis* Strain P2-1 Mediated Suppression of Carbendazim-Resistance Isolate of *Botryosphaeria dothidea*

To further test whether strain P2-1 had an antagonistic potential for fungicide-resistance isolate of *B*. *dothidea*, Bd7, a carbendazim-resistance isolate (Supplementary Figure 1; Wang L. et al., 2021), was antagonized with strain P2-1 bacterial suspension or supernatant. The strain P2-1 cell suspension and supernatant greatly reduced Bd7 mycelial growth in a dual culture study (Supplementary Table 3).

**FIGURE 5:**
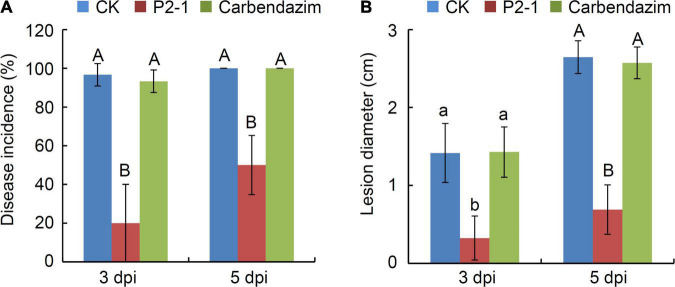
Effect of *B. velezensis* strain P2-1 cell suspension on apple ring rot caused by carbendazim-resistant isolate Bd7. **(A)** Effect of strain P2-1 cell suspension on apple ring rot disease incidence. Treatment with sterile distilled water or carbendazim was used as the negative control. **(B)** Effect of strain P2-1 cell suspension on apple ring rot disease lesion diameter. Each data represents the mean ± SD of three replicates. Letters above the bars indicate statistical significance and different lower case and upper case letters indicate significant different means at *p* < 0.05 and *p* < 0.01 based on Student’s *t*-test, respectively.

We also investigated the efficacy of strain P2-1 cell suspension in suppressing ring rot, caused by carbendazim-resistance isolate Bd7 in apple fruit. Strain P2-1 cell suspension dramatically reduced the incidence of disease and average lesion width in apple fruits, infected with Bd7 isolate (Figure 5). These findings revealed that strain P2-1 was capable of suppressing apple ring rot disease caused by the carbendazim-resistance isolate.

### Colonization of *Bacillus velezensis* Strain P2-1 in Apple Fruit Wounds

**FIGURE 6:**
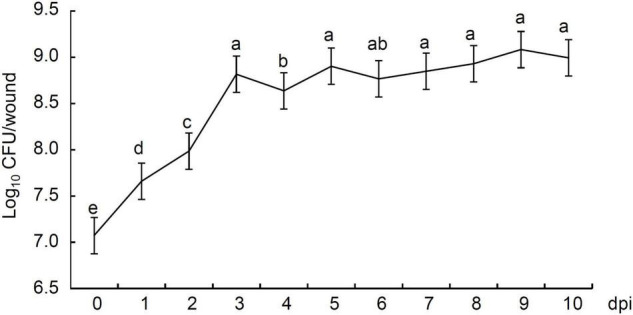
Population dynamics of *B. velezensis* strain P2-1 in wounds of apple fruit. The colonies were counted and population densities were expressed as log_10_ CFU/wound. Each data represents the mean ± SD of three biological replicates. Different letters indicate significant differences between different treatments according to Student’s *t*-test (*P* < 0.05).

We studied the population dynamics of strain P2-1 in apple fruit. Statistical results showed that the number of strain P2-1 colonies in inoculated apple fruit were 1.19 × 10^7^ CFU/wound at 0 dpi, and rapidly increased to 4.66 × 10^7^ CFU at 1 dpi (Figure 6). The population of strain P2-1 colonies reached a small peak with the population 6.61 × 10^8^ CFU at 3 dpi, and declined slightly at 4 dpi. Following that, the number of strain P2-1 slightly increased or decreased but remained typically consistent during the subsequent storage time. At 10 dpi, the population of strain P2-1 remained higher (9.93 × 10^8^ CFU) than the initial number (Figure 6). These results indicated the capability of strain P2-1 to successfully colonize in apple wounds.

### Efficacy of *Bacillus velezensis* Strain P2-1 for Reducing Apple Fruit Natural Decay

**FIGURE 7:**
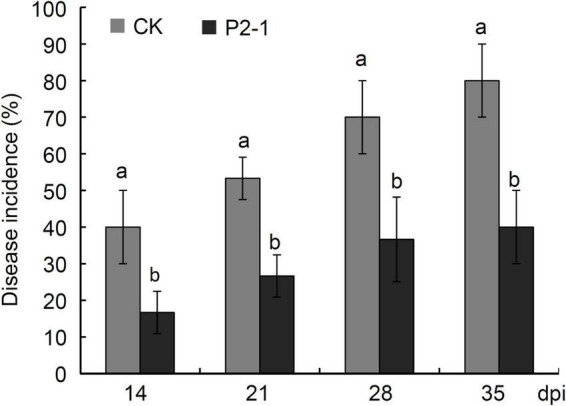
Disease incidence of natural decay in apple fruits treated with *B. velezensis* strain P2-1 cell suspension after harvest. Each data represents the mean ± SD of three biological replicates at each time point. Different letters indicate significant differences between different treatments according to Student’s *t*-test (*P* < 0.05).

Apples were treated with strain P2-1 cell suspension after harvest to examine the influence of strain P2-1 on apple decay during the postharvest period. Statistical results showed that strain P2-1 treatment significantly reduced the incidence of disease as compared to the control treatment during 35 days storage period (Figure 7). At 28 days and thereafter, the disease incidence of control treatment was more than 70%, while the disease incidence of strain P2-1 treatment remained less than 40% (Figure 7), suggesting that strain P2-1 was effective toward control of apple postharvest decay.

### Effects of *Bacillus velezensis* Strain P2-1 on Postharvest Quality Parameters of Apple

To assess the impact of antagonistic strain P2-1 on apple postharvest quality, four criteria were tested: (i) fruit firmness, (ii) ascorbic acid, (iii) titratable acid, and (iv) soluble sugar. The results indicated that strain P2-1 had no obvious influence on fruit firmness, ascorbic acid, titratable acid, or soluble sugar in apples (Table 2). This indicated that strain P2-1 had no significant influence on the postharvest quality of apple.

**TABLE 2:** Effects of *B. velezensis* strain P2-1 on postharvest quality parameters of apple.

Treatment	Firmness (N)	Ascorbic acid (mg/100 g)	Titratable acid (%)	Soluble sugar (%)
CK	31.62 ± 0.09	3.87 ± 0.58	0.19 ± 0.01	10.41 ± 0.37
P2-1	32.06 ± 0.94	3.68 ± 0.26	0.21 ± 0.01	11.05 ± 0.56

*Data represents the mean ± SD of three biological replicates. The samples treated with sterile distilled water were used as CK.*

### Analysis of Antibiotic Biosynthesis Genes From *Bacillus velezensis* Strain P2-1

**FIGURE 8:**
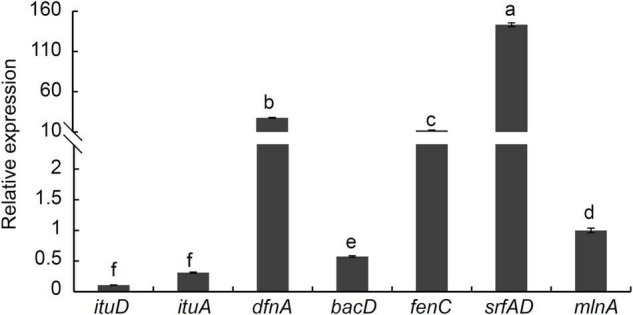
Analysis of antibiotic biosynthesis genes expression from *B. velezensis* strain P2-1 using qRT-PCR. Relative expression was determined by comparing *mlnA*, arbitrarily set to 1. Each data represents the mean ± SD of three biological replicates at each time point. Different letters indicate significant differences between different treatments according to Student’s *t*-test (*P* < 0.05).

*Bacillus* species are found to harbor numerous clusters of the genes involved in the synthesis of antifungal polyketides and lipopeptides. The PCR assays showed that the expected size of PCR products was obtained from strain P2-1 using specific primers (Supplementary Figure 2). Sequencing analysis results confirmed the involvement of eight genes in the lipopeptide biosynthesis including *ituD*, *ituA*, *srfAD*, *bacA*, *mlnA*, *bacA/B*, *bmyA*, and *dfnA*. Additionally, Figure 8 indicated that seven genes were expressed in strain P2-1, and *srfAD* and *dfnA* were expressed at a higher level upon comparison with other genes. The expression of *bymA* gene was not detected.

### Effect of *Bacillus velezensis* Strain P2-1 on Pathogenesis-Related Gene Expression in Apple Fruit

**FIGURE 9:**
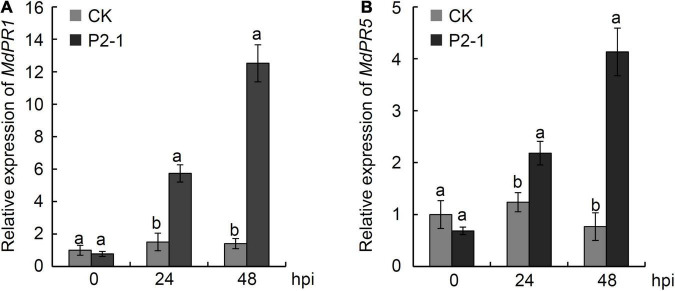
qRT-PCR assay for *MdPR1* gene **(A)** and *MdPR5* gene **(B)** expression in apple fruit induced by *B. velezensis* strain P2-1. Each data represents the mean ± SD of three biological replicates at each time point. Different letters indicate significant differences between different treatments according to Student’s *t*-test (*P* < 0.05).

To examine the expression of *PR* genes in response to strain P2-1 treatment, apple fruits were equally sprayed with strain P2-1 cell suspension and the levels of *MdPR1* and *MdPR5* genes were determined using qRT-PCR. The results indicated that strain P2-1 treatment considerably increased *MdPR1* and *MdPR5* expression when compared to the control. *MdPR1* transcript levels in strain P2-1-treated apple fruit reached their peak at 48 hpi, and they were 8.9-fold higher than in the control fruit (Figure 9A). The pattern of *MdPR5* gene expression was identical to that of *MdPR1*. *MdPR5* transcript levels were 5.4-fold greater in strain P2-1-treated apple fruit compared to the control (Figure 9B). This result indicated that strain P2-1 treatment could activate *PR* genes expression in apple fruit.

## Discussion

*B. dothidea*-caused ring rot is a serious postharvest disease that has an impact on global apple crop supplies. However, due to the latent infection characteristic, it is difficult to control this disease with chemical fungicides (Liu et al., 2011). Endophyte-based biocontrol of apple ring rot disease has emerged as a viable alternative to chemical fungicides in recent years (Chen et al., 2016; Zhang et al., 2016; Huang et al., 2021). In this study, we demonstrated that the use of *B. velezensis* strain P2-1 effectively reduced the width of apple ring rot lesions and the incidence of postharvest disease caused by *B. dothidea*, implying that this strain constitutes a promising resource for biocontrol of *B. dothidea* caused apple postharvest decay. In addition, *B. velezensis* strain P2-1 possessed broad-spectrum antagonistic activity against plant pathogens, which is consistent with previous studies (Khan et al., 2020; Liu et al., 2021).

Many plant phytopathogens, including *B. dothidea*, have been discovered to be resistant to fungicides (Zhou and Wang, 2001; Fan F. et al., 2016; Wang L. et al., 2021), which is one of the emerging challenges in plant disease management strategies. In recent research, we investigated the carbendazim sensitivity of *B. dothidea* isolates from apple orchards from nine Chinese provinces and discovered carbendazim-resistant isolates in three of them (Wang L. et al., 2021). An et al. (2016) also identified thiophanate-methyl resistant isolates in China. These findings encouraged us to emphasize the efficiency of endophytes in controlling fungicide-resistant *B. dothidea* isolates. Our results revealed that *B. velezensis* strain P2-1 cell suspension and supernatant could significantly inhibit the mycelial growth of carbendazim-resistant isolate. Moreover, *B. velezensis* strain P2-1 was found to be able to significantly suppress the apple ring rot disease caused by carbendazim-resistance isolate. This is the first report on the use of endophytes to prevent carbendazim resistance in *B. dothidea* isolates.

The ability of biocontrol agents to colonize and survive in host tissue is critical for their practical application. *B. velezensis* strain P2-1 was an endophytic bacterium isolated from the branches of apple. In addition, we could re-isolate strain P2-1 from the treated apple fruits. The population of strain P2-1 colonies reached a small peak at 3 dpi, which was 55.5-fold greater than the population at 0 dpi, and remained high throughout the subsequent storage period. The rapid growth of strain P2-1 in apple fruit wounds is necessary for its colonization and the production of antagonistic metabolites. When apple fruits were treated with strain P2-1, the natural decay disease incidence was greatly reduced as compared to the control treatment during storage. These findings indicate that *B. velezensis* strain P2-1 could colonize apple fruit well and was effective in preventing apple postharvest decay caused by *B. dothidea*.

Numerous strategies by which *Bacillus* spp. acts as a biocontrol agent against plant diseases have been described (Chen et al., 2016, 2020; Fan et al., 2017; Balderas-Ruíz et al., 2020; Liu et al., 2020). After antagonism with the *B. velezensis* strain P2-1, *B. dothidea* hyphae exhibited aberrant stretching, thin deformation, and a protoplast-like ball at the hyphae’s end (Figure 1B), which has been previously reported in *Botrytis cinerea* response to *B. velezensis* strain QSE-21 (Xu et al., 2021). Further research revealed that the supernatant of strain P2-1 exhibited potent antifungal activity toward *B. dothidea*, implying that the supernatant included antimicrobial substances. We confirmed that strain P2-1 harbored numerous gene clusters involved in the synthesis of antifungal lipopeptides and polyketides, such as *ituD*, *ituA*, *srfAD*, *bacA*, *mlnA*, *bacA/B*, *bmyA*, and *dfnA*. This finding is consistent with the previous studies conducted on other *Bacillus* species (Athukorala et al., 2009; Kim et al., 2017). We then examined the degree of expression of antibiotic biosynthesis genes and discovered that *srfAD* and *dfnA* were expressed at a higher level than other genes. This suggested that *srfAD* and *dfnA* in strain P2-1 may play an important role in antifungal activity against *B*. *dothidea*.

Biocontrol agents induced disease resistance is an effective method against plant diseases. Previous studies have mentioned that *Bacillus* spp. could enhance resistance against pathogens in fruits by inducing the transcription of defense-associated genes or enzymes (Waewthongrak et al., 2014; Wu et al., 2017; Huang et al., 2021; Wang F. et al., 2021). We also found a similar report that *B. velezensis* strain P2-1 treatment strongly elicited the *PR1* and *PR5* expressions in apple fruit.

The effect of fruit quality is one of the most important criteria in determining whether biocontrol agents may be used in the postharvest process in practice (Liu et al., 2010; Sun et al., 2017). Although some microorganisms have been identified as potential biocontrol agents against *B. dothidea* caused apple postharvest decay (Zhang et al., 2016; Huang et al., 2021), influence of antagonistic strains on the postharvest quality of apple has not yet been evaluated. In the present study, we noticed that *B. velezensis* strain P2-1 had no significant influence on the postharvest quality of apple fruit, which suggested the commercialization potential of *B. velezensis* strain P2-1, to control apple postharvest decay caused by *B. dothidea*.

## Conclusion

*B. velezensis* strain P2-1, isolated from apple branches, can successfully prevent apple postharvest decay caused by *B. dothidea* during storage, hence preserving postharvest quality. *B. velezensis* strain P2-1 harbored numerous clusters of the genes that are involved in the synthesis of antifungal lipopeptides and polyketides. Moreover, *B. velezensis* strain P2-1 treatment strongly induced *PR* genes expression in apple fruit. Overall, our research has identified a viable biocontrol agent for *B. dothidea*-induced apple postharvest decay and sheds light on the interaction mechanism between the biocontrol agent and pathogenic fungi.

## Data Availability Statement

The original contributions presented in the study are included in the article/Supplementary Material, further inquiries can be directed to the corresponding author/s.

## Author Contributions

HY designed the research and wrote the manuscript. HY, BS, and LW performed the experiments with helps from TH, ZZ, and HH. HY and HH analyzed the data. HT provided the funding. All authors contributed to the article and approved the submitted version.

## Conflict of Interest

The authors declare that the research was conducted in the absence of any commercial or financial relationships that could be construed as a potential conflict of interest.

## Publisher’s Note

All claims expressed in this article are solely those of the authors and do not necessarily represent those of their affiliated organizations, or those of the publisher, the editors and the reviewers. Any product that may be evaluated in this article, or claim that may be made by its manufacturer, is not guaranteed or endorsed by the publisher.

## Supplementary Material

Supplementary Figure 1 Test the carbendazim sensitivity of *B*. *dothidea* isolates. *B*. *dothidea* isolate Bd7 was used to value the carbendazim sensitivity. Carbendazim-sensitive isolate Bd220 was used as a negative control. The picture was taken at 5 dpi.

Supplementary Figure 2 PCR analysis of antibiotic biosynthesis genes from *B. velezensis* strain P2-1. M, Trans 2K DNA ladder, 1, *ituD*; 2, *ituA*; 3, *srfA*; 4, *baeA*; 5, *mlnA*; 6, *bacA/B*; 7, *bmyA*; 8, *dfnA*.
