# Supplementary figures and images for: Isolation and Characterization of Bacillus velezensis Strain P2-1 for Biocontrol of Apple Postharvest Decay Caused by Botryosphaeria dothidea
Source: Front Microbiol. 2022 Jan 4;12:808938. doi: 10.3389/fmicb.2021.808938 (PMC8764377; doi:10.3389/fmicb.2021.808938)

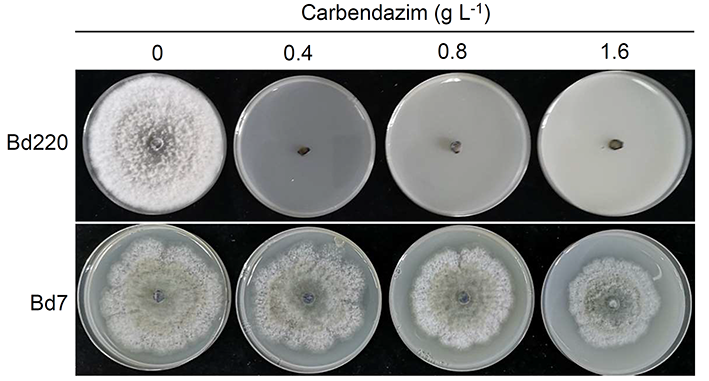

Supplement: Supplementary Figure 1 — Test the carbendazim sensitivity of B. dothidea isolates. B. dothidea isolate Bd7 was used to value the carbendazim sensitivity. Carbendazim-sensitive isolate Bd220 was used as a negative control. The picture was taken at 5 dpi. [file Image_1.TIF]

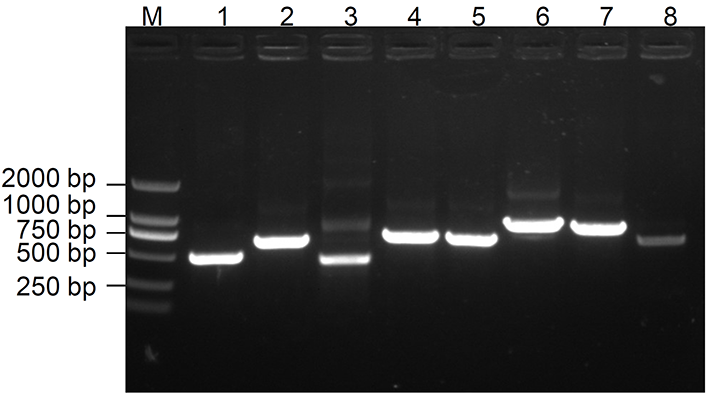

Supplement: Supplementary Figure 2 — PCR analysis of antibiotic biosynthesis genes from B. velezensis strain P2-1. M, Trans 2K DNA ladder, 1, ituD; 2, ituA; 3, srfA; 4, baeA; 5, mlnA; 6, bacA/B; 7, bmyA; 8, dfnA. [file Image_2.TIF]
