# Supplementary material for: Isolation and Characterization of Bacillus velezensis Strain P2-1 for Biocontrol of Apple Postharvest Decay Caused by Botryosphaeria dothidea
Source: Front Microbiol. 2022 Jan 4;12:808938. doi: 10.3389/fmicb.2021.808938 (PMC8764377; doi:10.3389/fmicb.2021.808938)
Supplement: Supplementary file 3 [file Table_1.DOCX]

**[Supplementary Table](https://www.frontiersin.org/articles/10.3389/fmicb.2021.659210/full#TS1)** **1.** Primers used in this study

| Gene | Primer | Primer sequence (5′-3′) | Reference |
| --- | --- | --- | --- |
| 16S rDNA | SR1 | AAGGAGGTGATCCAGCCGCA | Fan et al., 2016 |
|  | SR2 | AGAGTTTGATCCTGGCTCAG |  |
| gyrA | gyrA_F | CAGTCAGGAAATGCGTACGTCCTT | Rooney et al., 2009 |
|  | gyrA_R | CAAGGTAATGCTCCAGGCATTGCT |  |
| ropB | rpoB_2292f | GACGTGGGATGGCTACAACT |  |
|  | rpoB_3354r | ATTGTCGCCTTTAACGATGG |  |
| ituD (Iturin) | ITUD_F1 | TTGAAYGTCAGYGCSCCTTT | Kim et al., 2016 |
|  | ITUD_R1 | TGCGMAAATAATGGSGTCGT |  |
| ituA (Iturin A) | ITUD1F | GATGCGATCTCCTTGGATGT |  |
|  | ITUD1R | ATCGTCATGTGCTGCTTGAG |  |
| srf (Surfactin) | As1_F | CGCGGMTACCGVATYGAGC |  |
|  | Ts2_R | ATBCCTTTBTWDGAATGTCCGCC |  |
| baeA (Bacillaene) | baeR_F | ATGTCAGCTCAGTTTCCGCA |  |
|  | baeR_R | GATCGCCGTCTTCAATTGCC |  |
| mnl (Macrolactin) | mlnA_F | CCGTGATCGGACTGGATGAG |  |
|  | mlnA_R | CATCGCACCTGCCAAATACG |  |
| bacA/B (Bacilysin) | bacA/B_F | TGCTCTGTTATAGCGCGGAG |  |
|  | bacA/B_R | GTCATCGTATCCCACCCGTC |  |
| bmyA (Bacillomycin) | bmyA_F | CTCATTGCTGCCGCTCAATC |  |
|  | bmyA_R | CCGAATCTACGAGGGGAACG |  |
| dfnA (Difficidin) | dfnA_F | GGATTCAGGAGGGCATACCG |  |
|  | dfnA_R | ATTGATTAAACGCGCCGAGC |  |
| ituD | ituD-q_F | GCCATAGCTTAGGCGAATA | Kim et al., 2021 |
|  | ituD-q_F | GGAAAGTCTTCCGTCGATAC |  |
| ituA | ituA-q_F | CAAGCCTCTGGCGTTATATG |  |
|  | ituA-q_F | GTCAGCACATGCTCGATAAA |  |
| dfnA | dfnA-q_F | CCCATTACCTATGCCGAAAG |  |
|  | dfnA-q_F | GATCCGTTCGATACTCAATCC |  |
| bacD | bacD-F | TCGGTCGCGGTCATAAA |  |
|  | bacD-R | TGCCATCGGAGCGATAA |  |
| bmyA | bmyA-q_F | TCCAACCCGACCTTATGA |  |
|  | bmyA-q_F | CAATTCCCGGTACGTTAGAC |  |
| fen | fenC-q_F | CGGCTCCACTTTGTATATGG |  |
|  | fenC-q_F | GGACTTCCGCCAAGTAATC |  |
| srfA | srfAD-q_F | GGACACGGAACAAATCAAATG |  |
|  | srfAD-q_F | GGCTGAATGGCTGAGATG |  |
| mlnA | mnlA-q_F | CTCGGTGAAATGGACTGAAA |  |
|  | mnlA-q_F | CCTTCTCTTTCTCGCTTTCC |  |
| EF1α | MdEF1α-q_F | ACATTGCCCTGTGGAAGTT | Huang et al., 2021 |
|  | MdEF1α-q_F | GTCTGACCATCCTTGGAAA |  |
| PR1 | MdPR1-q_F | GCAGCAGTAGGCGTTGGTCCCT |  |
|  | MdPR1-q_F | CCAGTGCTCATGGCAAGGTTTT |  |
| PR5 | MdPR5-q_F | AGCAGCTTCCCTCCTCGGC |  |
|  | MdPR5-q_F | CCCAGAAGCGACCAGACC |  |

[**Supplementary Table**](https://www.frontiersin.org/articles/10.3389/fmicb.2021.659210/full#TS1) **2.** Physiological and biochemical characters of strain P2-1

| Test | Reaction^a^ |
| --- | --- |
| Gram stain | + |
| Voges-Proskauer (VP) test | + |
| Citrate | + |
| Methyl red test | + |
| V-general test | + |
| Nitrate reductase | + |
| Starch hydrolysis | + |
| Gelatin liquefaction | + |
| Glucose fermentation | + |
| Xylose fermentation | + |
| Mannitol fermentation | + |
| Growth |  |
| pH 5 | + |
| pH 7 | + |
| pH 8 | + |
| pH 9 | − |
| 20 ^o^C | + |
| 30 ^o^C | + |
| 40 ^o^C | + |
| 65 ^o^C | − |
| 2% NaCl | + |
| 5% NaCl | + |
| 7% NaCl | + |
| 10% NaCl | + |

^a^ + and − represent positive and negative reactions, respectively. Data within each column are the means of three independent experiments.

[**Supplementary Table**](https://www.frontiersin.org/articles/10.3389/fmicb.2021.659210/full#TS1) **3.** The inhibition of strain P2-1 against carbendazim-resistance isolate Bd7

| Treatment |  | Inhibition (%) |
| --- | --- | --- |
| P2-1 cell suspension |  | 70.7±3.8 |
| P2-1 cell-free supernatant | 1% | 28.4±1.4 |
|  | 2% | 59.8±2.7 |
|  | 5% | 75.3±3.9 |
|  | 10% | 89.7±1.5 |

Each data represents the mean ± SD of three biological replicates.
